# MaskUKF: An Instance Segmentation Aided Unscented Kalman Filter for 6D Object Pose and Velocity Tracking

**DOI:** 10.3389/frobt.2021.594583

**Published:** 2021-03-22

**Authors:** Nicola A. Piga, Fabrizio Bottarel, Claudio Fantacci, Giulia Vezzani, Ugo Pattacini, Lorenzo Natale

**Affiliations:** ^1^ Humanoid Sensing and Perception, Istituto Italiano di Tecnologia, Genova, Italy; ^2^ Dipartimento di Informatica, Bioingegneria, Robotica e Ingegneria dei Sistemi, Università di Genova, Genova, Italy; ^3^ Humanoid Sensing and Perception, Istituto Italiano di Tecnologia, Genova, Italy; ^4^ iCub Tech, Istituto Italiano di Tecnologia, Genova, Italy

**Keywords:** 6D object pose tracking, object velocity tracking, unscented Kalman filtering, deep learning-aided filtering, closed loop manipulation, humanoid robotics

## Abstract

Tracking the 6D pose and velocity of objects represents a fundamental requirement for modern robotics manipulation tasks. This paper proposes a 6D object pose tracking algorithm, called MaskUKF, that combines deep object segmentation networks and depth information with a serial Unscented Kalman Filter to track the pose and the velocity of an object in real-time. MaskUKF achieves and in most cases surpasses state-of-the-art performance on the YCB-Video pose estimation benchmark without the need for expensive ground truth pose annotations at training time. Closed loop control experiments on the iCub humanoid platform in simulation show that joint pose and velocity tracking helps achieving higher precision and reliability than with one-shot deep pose estimation networks. A video of the experiments is available as Supplementary Material.

## 1 Introduction

Object perception is one of the key challenges of modern robotics, representing a technology enabler to perform manipulation and navigation tasks reliably. Specifically, the estimation and tracking of the 6D pose of objects from camera images is useful to plan grasping actions and obstacle avoidance strategies directly in the 3D world. Object manipulation is another important application, where the evolution of the object state over time is fundamental for closing the control loop.

The problem of 6D object pose estimation and tracking has been extensively addressed both in the computer vision (Drost et al., 2010; Tjaden et al., 2017; Mitash et al., 2018) and robotics community, the latter focusing on solutions based on the use of Kalman and particle filtering techniques (Wüthrich et al., 2013; Issac et al., 2016; Vezzani et al., 2017; Deng et al., 2019). Recently, deep learning techniques have also been employed to solve the 6D object pose estimation problem (Li et al., 2018; Xiang et al., 2018; Wang C. et al., 2019). These methods, although being fairly complex and requiring a considerable amount of 3D labelled data, have shown impressive results in approaching the problem end-to-end. However, it is not clear whether their performance always generalizes to conditions not represented in the training set. Additionally, although successfully employed in one-shot grasping tasks (Wang C. et al., 2019), the possibility of closing the loop for tasks that require a continuous estimation of the object pose has not yet been thoroughly assessed.

In this paper we propose to take a step back from end-to-end deep pose estimation networks while still leveraging neural networks. We called our approach MaskUKF, as it uses 2D binary masks and an Unscented Kalman Filter (UKF) (Wan and Merwe, 2000) to track the pose and the velocity of the object given a 3D mesh model and depth observations in the form of point clouds.

Our contributions are the following:

• We combine consolidated techniques for object segmentation using deep neural networks with non-linear state estimation from the Kalman filtering literature.
• We show how to use depth information in the form of point clouds directly in an Unscented Kalman filtering setting and how to leverage recent advances in Serial Unscented filtering to reach real-time performance.
• We propose a heuristic 1-parameter procedure to identify and reject outliers from point clouds and increase tracking performance.
• We demonstrate the importance of joint pose and velocity tracking in a robotic setting with closed loop control experiments on a humanoid platform in simulation.

One of the main advantages of our approach consists of employing segmentation networks that are much simpler to train than end-to-end pose estimation architectures. Differently from other works, our approach does not require 6D labels of poses nor a specific training for each object of interest. Indeed, a suitable initial condition for the 6D pose of the object and a deep neural network (DNN) for segmentation, trained once for all the objects of interest, are enough to perform tracking. Finally, our approach allows estimating not only the pose of the object but also its velocity (linear and angular) that is paramount for closed loop control and in-hand manipulation (Viña et al., 2015).

We compare our technique against state-of-the-art pose estimation networks, object pose tracking algorithms, and a baseline consisting in a simpler architecture which uses an Iterative Closest Point (ICP) (Besl and McKay, 1992) registration algorithm from (Rusu and Cousins, 2011) and the masks from the segmentation network. Results demonstrate that we achieve and in most cases surpass the state-of-the-art and the baseline performance in terms of pose precision and frame rate. We further evaluate our algorithm in a closed loop scenario to demonstrate the advantages of our approach for robotic applications.

The rest of the paper is organized as follows. After a review of the state of the art on both 6D object pose estimation and tracking, we present our MaskUKF algorithm for 6D object pose tracking and the results of the experiments. Finally, we conclude the paper providing additional remarks and future perspectives.

## 2 Related Work

MaskUKF draws from state-of-the-art pose estimation techniques based on deep learning, as well as on classical techniques for tracking.

Recent works addressing the problem of 6D object pose tracking using classical tools (e.g., Kalman and particle filtering) focused on handling occlusion and outliers. Wüthrich et al. (2013) use depth measurements within an articulated particle filtering framework in order to explicitly model the occlusion of the object as a part of the state to be estimated. This information is then used to reject range measurements that do not belong to the surface of the object. Issac et al. (2016) propose a Robust Gaussian Filter for depth-based object-tracking that uses a heavy-tailed Gaussian noise model to counteract occlusions and outliers.

End-to-end deep architectures for object pose estimation have also been proposed. PoseCNN (Xiang et al., 2018) estimates the pose of the object in an end-to-end fashion from RGB images using a Convolutional Neural Network and refines it using depth information and a highly customized ICP. DenseFusion (Wang C. et al., 2019) incorporates segmentation, RGB and depth information in a dense pixel-wise fusion network whose output is refined using a dedicated iterative refinement network.

In contrast to the end-to-end approaches, PoseRBPF (Deng et al., 2019) combines Rao-Blackwellized particle filtering with a deep neural network in order to capture the full object orientation distribution, even in presence of arbitrary symmetries. However, state-of-the-art performance is achieved only with a large amount of particles, leading to low frame rates. Moreover, the method requires a separate training for each object. SegICP (Wong et al., 2017) follows a different path, incorporating off-the-shelf segmentation networks, a multi-hypothesis point cloud registration procedure and a Kalman filter to track the object pose and velocity.

In our work, we follow a similar path as SegICP (Wong et al., 2017) and PoseRBPF (Deng et al., 2019), since we combine DNNs with a filtering algorithm. In particular, we combine segmentation masks provided either by PoseCNN (Xiang et al., 2018) or Mask R-CNN (He et al., 2017) with an Unscented Kalman Filter. Differently from SegICP, we do not require an intermediate registration step between the segmentation module and the tracking stage. Instead, we directly design our filtering algorithm around the observed point cloud. In addition we also provide qualitative results on velocity tracking performance and we show the advantage of using it into a robotic application.

Our work is also similar to (Issac et al., 2016) in that we use a single Gaussian filter with depth information only for the actual tracking of the object and we explicitly take outliers into account. However, our outlier rejection procedure requires far less modifications of the standard Gaussian filter than (Issac et al., 2016) in order to be employed.

The works we have mentioned in this section support their findings by means of commonly adopted pose estimation metrics (Hodan et al., 2018) evaluated on standard or custom-made datasets. Our work is not different in this sense. Nevertheless, our aim is also to show that having acceptable performance according to such metrics does not necessarily hold when the output of the pose estimation/tracking is used for closed loop control in a robotic setting. To this end, we provide the results of experiments showing that joint pose and velocity tracking helps achieving higher precision and reliability in the execution of the task than with one-shot deep pose estimation networks or standard techniques like ICP.

## 3 Methodology

Given a sequence of input RGB-D images ${\left\{I_{t}\right\}}_{1\leq t\leq N_{I}}$, the task of 6D object pose tracking is to estimate the position $r_{t}$ and the orientation $o_{t}$ of the object O with respect to the camera frame, for every frame in the sequence. In our approach, we also estimate the relative velocity between the camera and object frames. The complete description of the object pose is then given by the state vector

$$x_{t}=\left(r_{t},v_{t},o_{t},{\dot{o}}_{t}\right)\in {\mathbb{R}}^{12},$$ (1)

where $r_{t}\in {\mathbb{R}}^{3}$ is the Cartesian position, $v_{t}\in {\mathbb{R}}^{3}$ is the Cartesian velocity, $o_{t}\in {\mathbb{R}}^{3}$ is the Euler ZYX representation of the orientation and ${\dot{o}}_{t}\in {\mathbb{R}}^{3}$ is the vector of the angular rates. Our approach relies on the assumption that an *instance segmentation* algorithm is available in order to identify the object of interest within the image $I_{t}$ and provide a segmentation mask $M_{t}$. The mask is used to extract a partial point cloud belonging to the visible part of the surface of the object from the depth map of the image $I_{t}$. The point cloud is then used as a measurement

$$y_{t}=\left(y_{t,1},\ldots ,y_{t,L}\right)\in {\mathbb{R}}^{3L},$$ (2)

in order to track the state of the object, with $y_{t,j}\in {\mathbb{R}}^{3}$ the *j*-th point and *L* the total number of points. Our approach makes the assumption that a 3D mesh model of the object O is available.

In order to track the state of the object over time, we decided to adopt an Unscented Kalman Filter (UKF) for the following reasons:

• the ability to handle non-linear relationships between state and measurements;
• the availability of a *serial* variant of the algorithm that efficiently processes high-dimensional measurements as in the case of point clouds;
• the possibility of integrating a motion model for the object in a principled manner;
• the recognized superiority (Wan and Merwe, 2000; Julier and Uhlmann, 2004) over alternatives based on linearization, e.g., the Extended Kalman Filter, in terms of unbiasedness and consistency.

In the remainder of this section, we describe MaskUKF in details.

### 3.1 Segmentation

The first step in the proposed pipeline requires to segment the object of interest within the input image $I_{t}$ in the form of a binary segmentation mask $M_{t}$. As the focus of our work is to develop a 6D object pose tracking algorithm, we rely on two existing segmentation architectures.

One is the semantic segmentation network taken from PoseCNN (Xiang et al., 2018), a recently proposed 6D pose estimation network. We used PoseCNN because it is also adopted in PoseRBPF (Deng et al., 2019) and DenseFusion (Wang C. et al., 2019), two algorithms we compare to in the experimental section of our work. In order to have a fair comparison, we adopted the same segmentation network.

The second architecture is Mask R-CNN (He et al., 2017), a consolidated region-based approach for object detection and segmentation. Differently from the segmentation network from PoseCNN, Mask R-CNN has not been proposed in the pose estimation and tracking communities and represents an off-the-shelf and commonly adopted solution for object segmentation. For this reason, we decided to test our method also with this segmentation framework.

### 3.2 Serial Unscented Kalman Filter

We model the belief about the state of the object *x* as the posterior distribution $p\left(x|y_{1:t}\right)$, given all the measurements $y_{1:t}$ up to the instant of time *t*. We adopt a Gaussian filtering framework and approximate $p\left(x|y_{1:t}\right)$ using a Gaussian distribution

$$\hat{p}\left(x|y_{1:t}\right)=N\left(x;\mu_{t},P_{t}\right),$$ (3)

under the hypothesis that the state *x* evolves according to a Markovian dynamic model, i.e.,

$$p\left(x_{t}|x_{1:\;t-1},y_{1:t-1}\right)=p\left(x_{t}|x_{t-1}\right),$$ (4)

and the measurements $y_{t}$ are conditionally independent given $x_{t}$, i.e.,

$$p\left(y_{t}|x_{1:\;t},y_{1:\;t-1}\right)=p\left(y_{t}|x_{t}\right).$$ (5)

We assume that the distributions in Eqs. 4 and 5 are Gaussian and have the following form

$$p\left(x|x_{t-1}\right)=N\left(x;f\left(x_{t-1}\right),Q_{t}\right),$$ (6)

$$p\left(y|x_{t}\right)=N\left(y;h\left(x_{t}\right),R_{t}\right),$$ (7)

where *f* and *h* are generic non-linear functions, $Q_{t}$ is referred as the process noise covariance matrix and $R_{t}$ as the measurement noise covariance matrix.

The probabilistic formulation in Eqs. 6 and 7 can be expressed, in functional form, in terms of the following motion model for the state *x* at time *t*

$$\begin{array}{l} x_{t}=f\left(x_{t-1}\right)+w_{t-1},\\ w_{t}∼N\left(0,Q_{t}\right), \end{array}$$ (8)

and measurement model for the measurement *y* at time *t*

$$\begin{array}{l} y_{t}=h\left(x_{t}\right)+\nu_{t},\\ \nu_{t}∼N\left(0,R_{t}\right). \end{array}$$ (9)

At each instant of time *t*, the previous belief

$$N\left(x;\mu_{t-1},P_{t-1}\right)$$ (10)

is propagated through the model in Eq. 8 in order to obtain the *predicted* mean $\mu_{t}^{-}$ and covariance $P_{t}^{-}$ of the state. Next, a new measurement $y_{t}$ is incorporated into the belief taking into account the measurement model in Eq. 9 according to the following *correction step*

$$\begin{array}{l} K_{t}=P_{xy,t}{\left(P_{y,t}\right)}^{-1},\\ \mu_{t}=\mu_{t}^{-}+K_{t}\left(y_{t}-{\hat{y}}_{t}\right),\\ P_{t}=P_{t}^{-}-K_{t}P_{xy,t}^{T} \end{array}$$ (11)

Here, $K_{t}$ is usually called the Kalman gain, $P_{xy,t}$ is the state-measurement covariance matrix, $P_{y,t}$ is the measurement covariance matrix and ${\hat{y}}_{t}$ is the predicted mean of the measurement. The actual estimate ${\hat{x}}_{t}$ is extracted as the mean $\mu_{t}$ of the approximate posterior $\hat{p}\left(x|y_{1:t}\right)$.

#### 3.2.1 The Unscented Transform Algorithm

The UKF algorithm, that we adopted, follows the general Gaussian filtering framework presented above. In the UKF, all the predicted and corrected mean and covariances are evaluated using the unscented transform (UT) algorithm (Julier and Uhlmann, 2004) that propagates the Guassian belief through the functions *f* and *h* even if they are not provided with an analytical expression (as in our case for the measurement function *h*, later described).

More precisely, the UT algorithm, which we recap here in the case of an additive transform y=g(x)+q, evaluates a Gaussian approximation of the joint distribution of *x* and *y*

$$\left(\begin{array}{c} x\\ y \end{array}\right)∼N\left(\left(\begin{array}{c} \mu_{x}\\ \mu_{y} \end{array}\right),\left(\begin{array}{cc} P_{x} & P_{xy}\\ P_{xy}^{T} & P_{y} \end{array}\right)\right),$$ (12)

when $x∼N\left(\mu_{x},P_{x}\right)$ and q∼N(0,Q) by means of a fixed number of so-called *sigma points* that capture the mean and covariance of the original distribution of (x,q) exactly. The main steps of the algorithm are as follows.

Let *n* be the size of the state *x*.

1) Form the *sigma points* for the random variable *x*:

$$\begin{array}{l} X^{\left(0\right)}=\mu_{x},\\ X^{\left(i\right)}=\mu_{x}+\sqrt{n+\lambda }{\left[\sqrt{P_{x}}\right]}_{i},\\ X^{\left(i+n\right)}=\mu_{x}-\sqrt{n+\lambda }{\left[\sqrt{P_{x}}\right]}_{i},\;i=1,\ldots ,n, \end{array}$$ (13)

where ${\left[A\right]}_{i}$ is *i*-th column of the matrix *A* and *λ* is a suitable constant.

2) Propagate the sigma points through the function *g*:

$$Y^{\left(i\right)}=g\left(X^{\left(i\right)}\right),\;i=0,\ldots ,2n.$$ (14)

3) Compute the propagated mean $\mu_{y}$, the propagated covariance $P_{y}$ and the cross-covarianace $P_{xy}$:

$$\begin{array}{l} \mu_{y}=\sum_{i=0}^{2n}W_{i}^{\left(m\right)}Y^{\left(i\right)},\\ P_{y}=\sum_{i=0}^{2n}W_{i}^{\left(c\right)}\left(Y^{\left(i\right)}-\mu_{y}\right){\left(Y^{\left(i\right)}-\mu_{y}\right)}^{T}+Q,\\ P_{xy}=\sum_{i=0}^{2n}W_{i}^{\left(c\right)}\left(X^{\left(i\right)}-\mu_{x}\right){\left(Y^{\left(i\right)}-\mu_{y}\right)}^{T}, \end{array}$$ (15)

with $W_{i}^{\left(m\right)}$ and $W_{i}^{\left(c\right)}$ suitable weights.

#### 3.2.2 Serial Correction Step

One challenge in the application of the correction step in Eq. 11 to our scenario is the necessity to invert the matrix $P_{y,t}$ having dimension 3L×3L. Here, *L*, the number of points within the point cloud, might be quite large (in the order of thousands or more), thus making difficult to perform the inversion of the matrix in real-time. An efficient solution to this problem, under the hypothesis that the noise covariance matrix $R_{t}$ in Eq. 9 is block diagonal, is given by the *serial* UKF correction step proposed in (McManus and Barfoot, 2011).

The serial processing approach reformulates the correction step in Eq. 11 in an algebrically equivalent way using the Sherman-Morrison-Woodbury identity (Jack and Morrison, 1950). The algebraic reformulation is designed around the following two matrices:

$$X_{t}=\left[\cdots \sqrt{W_{i}^{\left(c\right)}}\left(X_{t}^{\left(i\right),-}-\mu_{t}^{-}\right)\cdots \right]\;i=0,\ldots ,2n,$$ (16)

$$\begin{array}{c} Y_{t}=\left[\cdots \sqrt{W_{i}^{\left(c\right)}}\left(Y_{t}^{\left(i\right)}-{\hat{y}}_{t}\right)\cdots \right]\;i=0,\ldots ,2n\\ \;=\left[\begin{array}{c} Y_{t,1}\\ \cdots \\ Y_{t,L} \end{array}\right]. \end{array}$$ (17)

The matrix $X_{t}$ contains the weighted vector differences between the predicted sigma points $X_{t}^{\left(i\right),-}$ of the state, obtained after propagation through the model in Eq. 8, and the predicted mean of the state $\mu_{t}^{-}$. Similarly, the matrix $Y_{t}$ contains the weighted vector differences between the predicted sigma points $Y_{t}^{\left(i\right)}$ of the measurement, obtained after propagation through the model in Eq. 9, and the predicted mean of the measurement ${\hat{y}}_{t}$. This matrix can be also re-written in terms of the block matrices $Y_{t,j}\in {\mathbb{R}}^{3\times \left(2n+1\right)}$ where the *j*-th block contains three rows associated with the *j*-th point in the point cloud $y_{t}$.

Given the definition of $X_{t}$ and $Y_{t}$, it can be easily shown that the predicted covariance of the state $P_{t}^{-}$, the state-measurement covariance matrix $P_{xy,t}$ and the measurement covariance matrix $P_{y,t}$ in Eq. 11 can be expressed as

$$\begin{array}{l} P_{t}^{-}=X_{t}X_{t}^{T},\\ P_{xy,t}=X_{t}Y_{t}^{T},\\ P_{y,t}=Y_{t}Y_{t}^{T}+R_{t},\\ R_{t}=\text{diag}\left(R_{t,1},\cdots ,R_{t,L}\right) \end{array}$$ (18)

where $R_{t,j}\in {\mathbb{R}}^{3\times 3}$ is the measurement covariance matrix associated to the *j*-th point in the point cloud $y_{t}$. Substituting Eq. 18 in Eq. 11 results in the following re-formulation of the correction step:

$$\begin{array}{c} \mu_{t}=\mu_{t}^{-}+X_{t}Y_{t}^{T}{\left(Y_{t}Y_{t}^{T}+R_{t}\right)}^{-1}\left(y_{t}-{\hat{y}}_{t}\right),\\ P_{t}=X_{t}X_{t}^{T}-X_{t}Y_{t}^{T}{\left(Y_{t}Y_{t}^{T}+R_{t}\right)}^{-1}Y_{t}X_{t}^{T}\\ \;\;=X_{t}\left(I_{2n+1}-Y_{t}^{T}{\left(Y_{t}Y_{t}^{T}+R_{t}\right)}^{-1}Y_{t}\right)X_{t}^{T}. \end{array}$$ (19)

Using the Sherman-Morrison-Woodbury identity (Jack and Morrison, 1950) and the fact that $R_{t}$ is block diaognal, the covariance update equation in Eq. 19 becomes as follows:

$$\begin{array}{c} P_{t}=X_{t}{\left(I_{2n+1}+\sum_{j=1}^{L}Y_{t,j}^{T}R_{t,j}^{-1}Y_{t,j}\right)}^{-1}X_{t}^{T}\\ \;=X_{t}C_{t}^{-1}X_{t}^{T} \end{array}$$ (20)

where $C_{t}\in {\mathbb{R}}^{\left(2n+1\right)\times \left(2n+1\right)}$.

Following a similar reasoning, the state update equation in Eq. 19 becomes

$$\mu_{t}=\mu_{t}^{-}+X_{t}C_{t}^{-1}\left(\sum_{j=1}^{L}Y_{t,j}^{T}R_{t,j}^{-1}\left(y_{t,j}-{\hat{y}}_{t,j}\right)\right)$$ (21)

where $y_{t,j}\in {\mathbb{R}}^{3}$ is the *j*-th point extracted from the point cloud $y_{t}$ and ${\hat{y}}_{t,j}\in {\mathbb{R}}^{3}$ is the associated predicted mean extracted from the predicted measurement vector ${\hat{y}}_{t}$.

In summary, updating the state and the covariance of the state requires inverting the matrix $C_{t}$ having size (2n+1)×(2n+1) instead of the 3L×3L matrix $P_{y,t}$. Since the size *n* is typically much smaller than *L* (we recall in our case n=12), the serial approach requires to invert a relatively small matrix compared to the standard approach and allows achieving real-time computations.

### 3.3 Measurement Model

The specification of the measurement model accounts for the definition of the function *h* in Eq. 9. The role of *h* is to establish the relationship between the state $x_{t}$ and the measurements that we expect to observe when the object $O_{x_{t}}$ is in configuration $x_{t}$.

In this paper we adopt a *likelihood-field* like (Thrun et al., 2008) model and we decide to consider the point cloud $y_{t}$ as an ensemble of independent points

$$y_{t}=\left(y_{t,1},\ldots ,y_{t,L}\right)\in {\mathbb{R}}^{3L},$$ (22)

each distributed according to a normal distribution

$$y_{t,j}∼N\left(\pi_{t,j},\sigma_{j}^{2}I_{3}\right).$$ (23)

Given an object $O_{x_{t}}$, the mean $\pi_{t,j}$ is defined here as the point on the surface of the object $\partial O_{x_{t}}$ having minimum distance from the real measured point $y_{t,j}$. More formally,

$$\pi_{t,j}\left(x_{t}\right)=\text{arg}\underset{p\in \partial O_{x_{t}}}{\text{min}}\left\|y_{t,j}-p\right\|.$$ (24)

It can be easily shown that the model described in Eqs. 22–24 can be cast into the following measurement equation

$$\begin{array}{l} y_{t}=h\left(x_{t}\right)+\nu ,\\ \nu ∼N\left(0,R\right), \end{array}$$ (25)

where

$$\begin{array}{l} h\left(x_{t}\right)=\left(\pi_{t,1}\left(x_{t}\right),\ldots ,\pi_{t,L}\left(x_{t}\right)\right),\\ R=\text{diag}\left(\sigma_{1}^{2}I_{3},\ldots ,\sigma_{L}^{2}I_{3}\right). \end{array}$$ (26)

In summary, given the object in configuration $x_{t}$, the point cloud that we expect to observe is obtained by projecting the actual point cloud $y_{t}$ on the surface of the object. The resulting measurement Eq. 26 is non-linear with additive noise and the noise covariance matrix *R* has a diagonal block structure as required by the hypothesis of the serial UKF correction step.

The proposed measurement model also resembles the Nearest Neighbour procedure adopted in classical variants of the ICP registration algorithm. In addition to it, our measurement model provides a principled way to specify the uncertainty associated to the point cloud using the variances $\sigma_{j}^{2}$.

#### 3.3.1 Implementation of the Measurement Model

In order to implement the measurement equation Eq. 26, we should ideally solve the L×(2n+1) optimization problems corresponding to evaluation of *L* projections in Eq. 24 for each of the 2n+1 sigma points as per Eq. 14. In order to reduce the computational cost of these evaluations, we approximate the projections $\pi_{t,j}$ using a Nearest Neighbor approach.

In the following we assume that the 3D mesh of the object is available. First, we sample the mesh using the Poisson Disk Sampling algorithm proposed in White et al. (2007). This produces a cloud PC of uniformly distributed points about the surface of the object that we represent using a 3D k-d tree *T*. Then, in order to evaluate an approximation of the projections $\pi_{t,j}\left(X_{t}^{\left(i\right)}\right)$ for a given sigma point $X_{t}^{\left(i\right)}$, it is sufficient to express the measurement $y_{t,j}$ in the reference frame of the object in configuration $X_{t}^{\left(i\right)}$ and to perform an efficient Nearest Neighbour search on the k-d tree *T* for each transformed point $y_{t,j}\left(X_{t}^{\left(i\right)}\right)$ resulting in the propagated sigma point $Y_{t}^{\left(i\right)}\left(X_{t}^{\left(i\right)}\right)$. The propagated sigma points, expressed in the reference frame of the object, are finally converted back in the reference frame of the camera obtaining the sigma points $Y_{t}^{\left(i\right)}$.

We stress the fact that our choice to project the measurements onto the surface of the object in a given configuration represents a possible, although not unique, solution to the data association problem between the measurements $y_{t}$ and the points PC sampled on the mesh of the object. This solution guarantees that the length of each propagated sigma point $Y_{t}^{\left(i\right)}$ is the same as the vector of the measurement $y_{t}$, i.e., 3L, and allows evaluating the mean $\mu_{y}$ and the associated quantities in Eq. 15. Different solutions, e.g., projecting the points on the sampled cloud PC that are visible from the camera viewpoint onto $y_{t}$, would possibly produce sigma points of incompatible sizes thereby making impossible to execute the UT algorithm.

We also remark that the implementation of the projections $\pi_{t,j}$ using a Nearest Neighbour search is made possible by the adoption of the Unscented Kalman filter that, differently from other alternatives such as the Extended Kalman filter, does not require the evaluation of the Jacobian of the projections, i.e., of the Nearest Neighbour search, with respect to the state *x* which might be intractable.

### 3.4 Outlier Rejection

Point clouds from modern RGB-D sensors are characterized by non Gaussian noise and outliers that violate the hypotheses of the model in Eq. 25 leading to divergent estimates in Gaussian filters (Issac et al., 2016). To tackle this, we try to identify outliers by picking pairs of points on the point cloud $y_{t}$, $p_{i}$ and $p_{j}$, respectively, and their projections, $\pi_{i}$ and $\pi_{j}$, on the surface of the object in configuration ${\hat{x}}_{t-1}$. Under the assumption that the tracking process has already reached a steady state condition, we expect that the point cloud at time *t* fits very closely the surface of the object in the estimated configuration at time t−1. As a result, the distance $d_{ij}$ between the two points and the distance $d_{ij}^{\pi }$ between their projections should be preserved if both $p_{i}$ and $p_{j}$ are not outliers, i.e., belongs to the surface of the object. Then, by comparing the absolute difference between $d_{ij}$ and $d_{ij}^{\pi }$ to a threshold

$$\left|d_{ij}-d_{ij}^{\pi }\right|>\delta ,$$ (27)

we are able to identify possible outliers. In such a case, the point between $p_{i}$ and $p_{j}$ that has higher distance from its projection is marked as an outlier.

Since outliers that violate the additive Gaussian noise hypothesis are usually distributed relatively far from the actual surface of the object, we propose, as a heuristic, to choose candidate pairs of points as the combination of one point $p_{i}$ and its furthest point $p_{i}^{f}$ on the point cloud for each point on the point cloud $y_{t}$. Every time an outlier is found, it is removed from the point cloud and the procedure is repeated until all points have been visited.

An efficient evaluation of the points $p_{i}^{f}$ is obtained using the algorithm proposed in Curtin and Gardner (2016).

### 3.5 Motion Model

The motion model in Eq. 8 accounts for our prior knowledge about the motion of the object. We use the White Noise Acceleration model (WNA) (Bar-Shalom et al., 2002) from the tracking literature. This model assumes that the Cartesian acceleration $\ddot{r}\left(t\right)$ is driven by a white noise process $w_{r}\left(t\right)$ with assigned power spectral density $Q_{r}\in R^{3\times 3}$. The same model has been adopted for the rotational part of the state, with power spectral density $Q_{o}$, resulting in

$$\begin{array}{l} \ddot{r}\left(t\right)=\omega_{r}\left(t\right),\\ \ddot{o}\left(t\right)=\omega_{o}\left(t\right). \end{array}$$ (28)

In order to obtain a discrete time model as in Eq. 8, we exactly discretized the WNA model assuming constant Cartesian and angular Euler rates within each sampling period ${\text{Δ}}_{\text{T}}$. The final model is as follows

$$\begin{array}{l} x_{t}=Fx_{t-1}+w,\\ w∼N\left(0,Q\right), \end{array}$$ (29)

where *F* is the state transition matrix, that depends on $\Delta_{T}$, and *Q* is the process noise covariance matrix depending on $Q_{r}$, $Q_{o}$ and ${\text{Δ}}_{T}$. The complete expressions of the matrices *F* and *Q* can be found in Bar-Shalom et al. (2002).

### 3.6 6D Object Pose Tracking Framework

**FIGURE 1:**
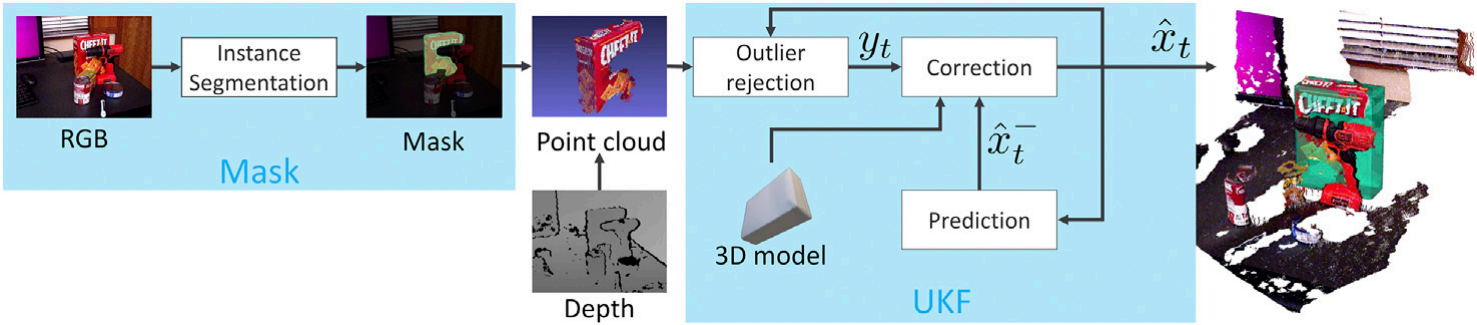
Illustration of our MaskUKF framework for 6D object pose tracking. For each RGB-D frame, a generic instance segmentation algorithm provides the segmentation mask of the object of interest. The mask is combined with the depth frame to produce a partial point cloud of the object that is further refined to remove possible outliers. The resulting measurements are fed into an Unscented Kalman filter to update the belief of the object pose and velocity.

The tracking process is initialized by specifying the initial mean $\mu_{0}$ and covariance $P_{0}$ representing the initial belief on the state of the object. Then, the tracking process starts as soon as the first segmentation mask $M_{0}$ is available. At each frame $I_{t}$ we apply the binary mask $M_{t}$ to the depth map and extract the segmented point cloud $y_{t}$, refining it as described in Section 3.4 to remove outliers. The resulting point cloud is used to correct our belief of the pose and velocity of the object. Our 6D object pose tracking framework is shown in Figure 1.

In case the mask is not available at time *t*, we use the most recent available mask instead. Differently from pose estimation networks, such as (Wang C. et al., 2019), this choice allows our method to run at full frame rate even when coupled with slower instance segmentation algorithms. If $M_{t}$ is empty, due to total occlusion, or when most of the extracted 3D points are invalid, due to lack of texture, we stop feeding the measurements $y_{t}$ to the correction step in Eqs. 20 and 21 because there are none or too few to be informative enough. Usually, such an absence of measurements is counteracted by the usage of the Kalman prediction step only. However, if the object is undergoing a motion characterized by moderate to high velocities, this approach is discouraged and leads to unbounded predictions. We instead keep performing the correction step using a static virtual point cloud ${\tilde{y}}_{t}\left({\hat{x}}_{t-1}\right)$ sampled on the surface of the object in a configuration corresponding to the last estimate available. As a result, the estimated velocities are driven to zero in case the absence of information persists for multiple frames allowing the tracking process to safely recover when the measurements are available again. We remark that the proposed procedure does not require to manually force the state of the filter which would impair the correctness of the state covariance associated with the state.

## 4 Experiments

In this section we present the results of two separate sets of experiments that we conducted in order to evaluate the performance of our method.

In the first set of experiments, we evaluate our method against a standard dataset for pose estimation benchmarking. Following prior work (Li et al., 2018; Wang C. et al., 2019; Deng et al., 2019), we adopt the YCB Video dataset (Xiang et al., 2018). The main goals of the first set of experiments are to:

• compare the proposed architecture against state-of-the-art algorithms in tracking, i.e., PoseRBPF (Deng et al., 2019), and pose estimation, i.e., DenseFusion (Wang C. et al., 2019), from RGB-D images using standard metrics from the computer vision and tracking communities;
• assess the advantage of the proposed architecture as opposed to the Iterative Closest Point (ICP) baseline.

Moreover, these experiments aim to:

• determine the performance of the proposed approach in multi-rate configuration, when segmentation masks are available at low frequency, i.e., at 5 fps, as it might happen in a real scenario;
• assess the ability of our method to estimate the 6D velocity of the object (linear and angular);
• demonstrate the effectiveness of the outlier rejection procedure described in Section 3.4.

In the second set of experiments, we compare the performance of the proposed architecture in a robotic setting against a one-shot pose estimation network, i.e., DenseFusion (Wang C. et al., 2019), and against ICP within a dynamic pouring experiment carried out in the Gazebo (Koenig and Howard, 2004) environment using the iCub (Metta et al., 2010) humanoid robot platform. In these experiments, we employ a robotic control system in order to track a bowl container with the end-effector of the robot during a pouring action. We close the loop using either the output of our method or the output of the methods we compare to. The aim of these experiments is to understand how different algorithms for object pose estimation and tracking affect the closed loop tracking performance and the reliability of the simulated system in a dynamical robotic setting. Our results include end-effector tracking errors for several configurations of the control gains as well as the qualitative evolution of the end-effector trajectories over time.

Overall, the aim of our experiments is twofold. On one hand, we want to test our method on a standard computer vision dataset that has been adopted in the pose estimation and tracking communities. On the other hand, we want to test the effectiveness of our method in a robotic scenario involving closed loop vision-based control.

### 4.1 Comparison with the State of the Art

In order to conduct our experiments, we considered several algorithms from the state of the art.

PoseRBPF (Deng et al., 2019) is a 6D object pose tracking algorithm adopting a deep autoencoder for implicit orientation encoding (Martin et al., 2018) and a particle filter to track the position and the orientation of the object over time from RGB-D observations. This algorithm requires a textured 3D mesh model of the object both at training and test time and requires a 2D detector in order to be initialized. The authors adopt the segmentation stage of PoseCNN as source of detections. In order to have a fair comparison with PoseRBPF, we also adopted PoseCNN as source of segmentation masks in our experiments.

Following the authors of PoseRBPF, we also compare our method with a 6D object pose estimation network from the state of the art, DenseFusion (Wang C. et al., 2019). This network fuses segmentation, RGB and depth information at the pixel level in order to regress the 6D pose of the object. An iterative refinement network is used to refine the pose. We notice that this method has similar requirements to our since it needs the object segmentation in order to isolate 2D and 3D features on the part of the input image containing the object. Moreover, it requires a 3D mesh model of the object of interest in order to be trained. DenseFusion adopts PoseCNN as source of segementation masks as our method.

As baseline, we also compare against a basic point cloud registration ICP algorithm (Rusu and Cousins, 2011). In order to have a fair comparison with our pipeline, we adopted an ICP algorithm that is assisted by segmentation and that is equipped with its own outlier rejection procedure. Given that the ICP algorithm needs to be initialized at each iteration, we adopted the estimate from the previous frame as the initialization. Similarly to the other algorithms that we considered, the ICP algorithm requires a 3D mesh model of the object in order to sample a target point cloud, on the surface of the object, to be registered with the point cloud extracted from the depth image.

In summary, our method and all the methods that we considered use the RGB data in order to segment or detect the object in the image plane at initialization time or at each frame and use the depth information as observation to track or estimate the pose of the object. Moreover, they all require a 3D mesh model at training time or at execution time.

Even though our approach is somewhat similar to the SegICP (Wong et al., 2017) algorithm, we do not compare against it as the authors do not provide their results on the YCB Video Dataset and the implementation of the algorithm is not publicly available.

### 4.2 Implementation Details

In order to perform our experiments we used either the semantic segmentation network from the PoseCNN (Xiang et al., 2018) framework as trained by the authors on all the 200k+ frames of the YCB Video dataset or the Mask R-CNN (He et al., 2017) model that we trained using 50k frames from the same dataset. For the experiments in the Gazebo environment, we relied on a simulated segmentation camera.

The initialization of the tracking is not a focus of this paper. For this reason, we reset both the UKF and the ICP to the ground truth perturbed with a displacement of 5 cm along each Cartesian axis and a variation of 10° for each Euler angle. Although we could have initialized the algorithms using a pose estimation network, to the best of our knowledge, the magnitude of our displacement is higher than the error produced by state-of-the-art pose estimation algorithms (this is actually confirmed by our Table 1, e.g., for DenseFusion for the <2 cm metric). Hence, our choice poses a even more challenging condition than initializing using a pose estimation network. We stress that the initialization is done as soon as the object is visible in the scene and the first segmentation mask is available. Differently from other state-of-the-art tracking algorithms, e.g., PoseRBPF (Deng et al., 2019), we never re-initialize our algorithm within a video sequence, e.g., after a heavy occlusion of the object.

**TABLE 1:** Quantitative evaluation of 6D pose on YCB-Video Dataset key-frames (ADD-S). We do not report ADD-S < 2 cm for PoseRBPF as this information is not available in the original paper. In this experiment, segmentation masks are available at each frame $I_{t}$. A bold entry indicates a strictly better result (i.e. if different algorithms have the same best result, the associated entries are not bolded).

	Pose RBPF	DenseFusion (iterative)		ICP		MaskUKF		ICP		MaskUKF	
Segm.	-	PoseCNN						Mask R-CNN			
Objects	AUC	AUC	<2 cm	AUC	<2 cm	AUC	<2 cm	AUC	<2 cm	AUC	<2 cm
002	95.1	**96.4**	100.0	95.0	100.0	96.1	99.6	93.9	97.6	96.1	99.6
003	93.0	95.5	99.5	**97.0**	**100.0**	96.7	99.7	96.3	**100.0**	89.2	82.3
004	95.5	97.5	100.0	97.6	100.0	**98.1**	99.6	97.0	99.3	**98.1**	99.6
005	93.8	94.6	96.9	94.3	**97.4**	**96.7**	96.9	95.8	96.8	94.1	91.3
006	96.3	97.2	100.0	97.8	100.0	**98.2**	99.4	98.0	100.0	**98.2**	99.4
007	95.3	96.6	100.0	96.9	99.8	94.1	94.6	**97.2**	100.0	96.2	99.7
008	92.0	**96.5**	100.0	91.0	100.0	93.8	98.1	91.0	95.8	75.7	43.5
009	97.5	98.1	100.0	97.7	100.0	98.3	99.5	98.5	100.0	**98.7**	99.5
010	77.9	91.3	93.1	87.5	84.6	93.2	**96.3**	84.1	83.0	**94.0**	93.9
011	86.9	96.6	100.0	97.2	100.0	97.6	99.7	96.6	97.6	**97.7**	99.7
019	94.2	97.1	100.0	**97.7**	100.0	96.5	99.3	97.6	100.0	96.4	99.3
021	93.0	95.8	100.0	94.9	99.2	**97.0**	99.6	96.1	100.0	**97.0**	99.6
024	94.2	88.2	98.8	97.3	**100.0**	**97.6**	99.5	94.2	89.9	97.5	99.5
025	97.1	97.1	100.0	94.5	100.0	97.1	99.7	94.5	100.0	**97.4**	99.7
035	96.1	96.0	98.7	**97.5**	**100.0**	96.5	98.9	97.4	99.8	89.8	78.8
036	89.1	89.7	94.6	92.4	99.6	**95.0**	99.6	91.7	97.9	94.7	99.6
037	85.6	95.2	100.0	84.8	64.1	**96.5**	99.4	86.0	64.1	95.7	100.0
040	97.1	**97.5**	100.0	91.5	94.4	96.8	100.0	91.1	94.4	95.6	98.8
051	**94.8**	72.9	**79.2**	53.9	54.8	70.0	71.9	43.1	43.7	47.1	42.1
052	**90.1**	69.8	**76.3**	76.1	62.5	80.3	71.8	80.8	60.1	35.7	14.4
061	95.7	92.5	100.0	84.1	40.3	**97.7**	99.7	93.6	100.0	97.1	99.7
ALL	93.3	93.1	**96.8**	91.9	92.7	**94.2**	95.9	91.5	92.5	89.5	87.8

We remark that all the results presented hereafter are obtained from a single run of our method since both the UKF and the ICP are deterministic.

To enforce repeatability in the evaluation of the projections in Eq. 24, we relied on uniform point clouds sampled on the 3D mesh of the objects as provided within the YCB Video Dataset.

The code used for the experiments is made publicly available for free with an Open Source license online.^1^

### 4.3 Results on YCB Video Dataset

#### 4.3.1 Description of the YCB Video Dataset

The YCB Video dataset features RGB-D video sequences of 21 objects from the YCB Object and Model Set (Calli et al., 2015) under different occlusion conditions. The dataset contains 92 RGB-D video sequences recorded at 30 fps, split in a training set of 80 videos and a testing set of 12 videos from which 2,949 key-frames are extracted in order to evaluate performance metrics. Every frame is annotated with segmentation masks and 6D object poses.

#### 4.3.2 Evaluation Metrics

We use two different metrics. The first metric is the average closest point distance (ADD-S) (Xiang et al., 2018) which synthesizes both symmetric and non-symmetric objects into an overall evaluation. This metric considers the mean distance between each 3D model point transformed by the estimated pose and its closest neighbour on the target model transformed by the ground truth pose. Following prior work (Wang C. et al., 2019), we report, for each object, the percentage of ADD-S smaller than 2 cm and the area under the ADD-S curve (AUC) with maximum threshold set to 10 cm.

We report results using the ADD-S metric, because it allows comparing with other state-of-the-art algorithms especially when their implementation is not publicly available. Indeed, the authors of PoseRBPF (Deng et al., 2019) and DenseFusion (Wang C. et al., 2019) report the results of their experiments using this metric. Nevertheless, the ADD-S metric does not report the actual error in position and orientation in the algorithm output that we deem very important in order to evaluate an algorithm for robotic applications. Moreover, the ADD-S metric is evaluated on a subset of the video frames, called key-frames. For these reasons and for a better evaluation, we also report, as additional metric, the Root Mean Square Error (RMSE) *over the entire trajectory* of the Cartesian error $e_{r,t}$ and the angular error $e_{o,t}$, which is the standard metric used in tracking literature (Bar-Shalom et al., 2002). We evaluate the angular error as the geodesic (Huynh, 2009) between the actual and the estimated orientation in [0,π). In order to have a fair comparison, we omitted part of the angular error due to symmetries for texture-less objects and for objects that have a pure cylindrical shape. In fact, algorithms that only use the depth information, as ours and ICP, cannot infer the rotation along the main axis of rotation of such objects.

#### 4.3.3 ADD-S Metric

Table 1 shows the evaluation for all the 21 objects in the YCB Video dataset. We report the ADD-S AUC (<10 cm) and the ADD-S < 2 cm metrics evaluated on the 2,949 key-frames provided. In order to evaluate on the exact key-frames, we run our experiments under the hypothesis that the segmentation results are available for each frame $I_{t}$.

When considering segmentation from PoseCNN, the percentage of frames with error less than 2 cm is lower for our method than DenseFusion. However, our method outperforms both the DenseFusion framework and ICP in terms of the area under the ADD-S curve with maximum threshold set to 10 cm. This demonstrates that, in the interval [0 cm, 2 cm), errors for MaskUKF have a distribution that is more concentrated towards zero.

We remark that even if the increase in performance is moderate, our architecture is much simpler than some pose estimation networks, such as DenseFusion, especially in terms of training requirement (we recall that MaskUKF can track object poses given a suitable initial condition and a trained segmentation network). Our method also outperforms the tracking algorithm PoseRBPF under the same metric.

We detected a performance drop when comparing results obtained with different segmentation networks, namely PoseCNN and Mask R-CNN. We verified that this is due to missing detections or completely wrong segmentation from Mask R-CNN. See, e.g., objects 003, 008, 035. Another example is that of the two “clamps” (051 and 052) that have the very same shape but a different scale, hence difficult to segment using a conventional instance segmentation network. For these two objects PoseRBPF outperforms all the other algorithms. More in general, the results suggest that the performance might vary with the specific segmentation algorithm that is adopted. In this work, differently from PoseRBPF and DenseFusion that rely on PoseCNN for the detection and segmentation, respectively, we have also considered a general purpose instance segmentation algorithm, such as Mask R-CNN, that has not been trained in the context of 6D pose estimation.

#### 4.3.4 RMSE Metric

In Table 2, we report the RMSE metrics evaluated on *all the frames*. In order to compare with DenseFusion, we run their pipeline on all the frames of each video sequence. We cannot compare with PoseRBPF since their implementation is not publicly available.

**TABLE 2:** Quantitative evaluation of 6D pose on all frames of YCB-Video Dataset (RMSE). Objects in bold are considered symmetric. In this experiment, segmentation masks are available at each frame $I_{t}$. A bold entry indicates a strictly better result (i.e. if different algorithms have the same best result, the associated entries are not bolded).

	Dense Fusion (iterative)		ICP		MaskUKF		ICP		MaskUKF	
Segm.	PoseCNN						Mask R-CNN			
Objects	$e_{r}\;\left(\text{cm}\right)$	$e_{o}\;\left({}^{\circ}\right)$	$e_{r}\;\left(\text{cm}\right)$	$e_{o}\;\left({}^{\circ}\right)$	$e_{r}\;\left(\text{cm}\right)$	$e_{o}\;\left({}^{\circ}\right)$	$e_{r}\;\left(\text{cm}\right)$	$e_{o}\;\left({}^{\circ}\right)$	$e_{r}\;\left(\text{cm}\right)$	$e_{o}\;\left({}^{\circ}\right)$
**002**	**0.464**	2.35	0.742	2.05	0.592	1.63	1.11	14.8	0.599	**1.55**
003	0.918	4.86	**0.41**	**2.31**	0.661	2.96	0.712	3.12	3.59	59.7
004	**0.334**	2.93	0.406	2.02	0.398	**1.84**	0.676	76.1	0.506	2.02
**005**	**0.47**	9.04	1.56	63.7	1.15	13	1.53	45.6	3.24	**6.32**
006	0.449	27.8	0.356	5.28	0.46	3.3	**0.249**	3.63	0.34	**3.15**
**007**	**0.447**	37.2	0.603	61.6	1.26	**29.5**	0.475	61.3	0.772	77.1
008	**0.651**	**7.61**	1.4	89.6	1.49	18.3	1.87	20.1	8.92	142
009	0.3	3.88	0.559	4.56	0.458	2.39	**0.291**	2.01	0.411	**1.82**
010	7.43	53.9	4.12	131	**1.63**	68.5	4.93	114	2.21	**5.62**
011	0.678	46.1	**0.652**	10.6	0.937	5.52	1.54	19.4	0.868	**5.29**
019	0.297	7.56	**0.225**	**2.13**	0.866	3.97	0.237	2.3	0.885	4.04
021	1.05	14.2	1.36	87	0.556	7.5	0.803	24.4	**0.535**	**6.98**
**024**	0.705	85.9	**0.45**	4.2	0.513	**2.74**	1.35	25	0.492	3.14
025	0.659	50.3	0.492	122	0.567	**6.01**	0.497	120	**0.468**	17.4
035	0.559	22.1	0.543	**3.35**	1.1	6.1	**0.506**	3.75	3.21	21.1
**036**	1.64	7.69	1.8	9.49	**1.41**	**1.63**	3.01	21.3	1.55	1.74
037	1.63	82.3	2.81	31	**0.634**	**5.08**	3.19	32.4	1.3	5.83
**040**	**0.464**	**4.35**	2.75	10.8	0.962	5.51	2.78	120	1.77	6.69
**051**	**12.1**	98.1	15.3	75.8	13.2	85.2	17.9	74.8	16.7	**72.8**
**052**	4.75	114	5.82	52.3	5.45	**22.8**	**4.28**	24.3	13.5	67.8
061	0.895	91.6	4.26	139	**0.485**	**3.53**	2.29	176	0.68	10.5
ALL	3.23	44.8	3.69	60.8	**3.07**	**26**	4.13	62.3	4.9	38.5

When equipped with the PoseCNN segmentation network, our method outclasses both the DenseFusion framework and ICP. While the increase in performance is minimal for the Cartesian error, the difference is considerable for the angular error that, in average, is almost halved with respect to the competitors.

Using segmentation from Mask R-CNN, our method still outperforms ICP in terms of the angular error while the Cartesian error is comparable. As already noticed, also the RMSE metric reveals, on average, a drop in performance when using this segmentation with respect to PoseCNN due to missing detections or completely wrong segmentation.

If we consider both segmentations, we can see that, for some specific objects, the reduction in angular error is indeed substantial. As an example, when using Mask R-CNN, the angular error for object 005 is 6.32° for MaskUKF while, for ICP, is 45.6°. With the same segmentation, the error for object 010 is 5.62° while, for ICP, is 114°. Moving to the PoseCNN segmentation, for the object 061 MaskUKF achieves 3.53° while, for DenseFusion, the error is 91.6° and, for ICP, it is even higher, 139°. Similar examples are those of objects 021, 024 and 037. As can be seen from Table 3, where we reported the maximum percentage of occlusion for each object among all YCB Video frames, these objects are among those involved in moderate to severe occlusions.

**TABLE 3:** Maximum occlusion percentage among YCB Video Dataset frames for each object.

Objects	%
005_tomato_soup_can	96.9
010_potted_meat_can	69.6
061_foam_brick	68.2
003_cracker_box	61.2
024_bowl	59.7
052_extra_large_clamp	59.0
008_pudding_box	51.4
035_power_drill	47.6
037_scissors	45.4
036_wood_block	44.9
002_master_chef_can	44.6
021_bleach_cleanser	43.0
011_banana	42.5
004_sugar_box	40.95
051_large_clamp	39.3
040_large_marker	22.3
025_mug	15.5
019_pitcher_base	12.8
007_tuna_fish_can	8.0
006_mustard_bottle	3.6
009_gelatin_box	0.8

**FIGURE 2:**
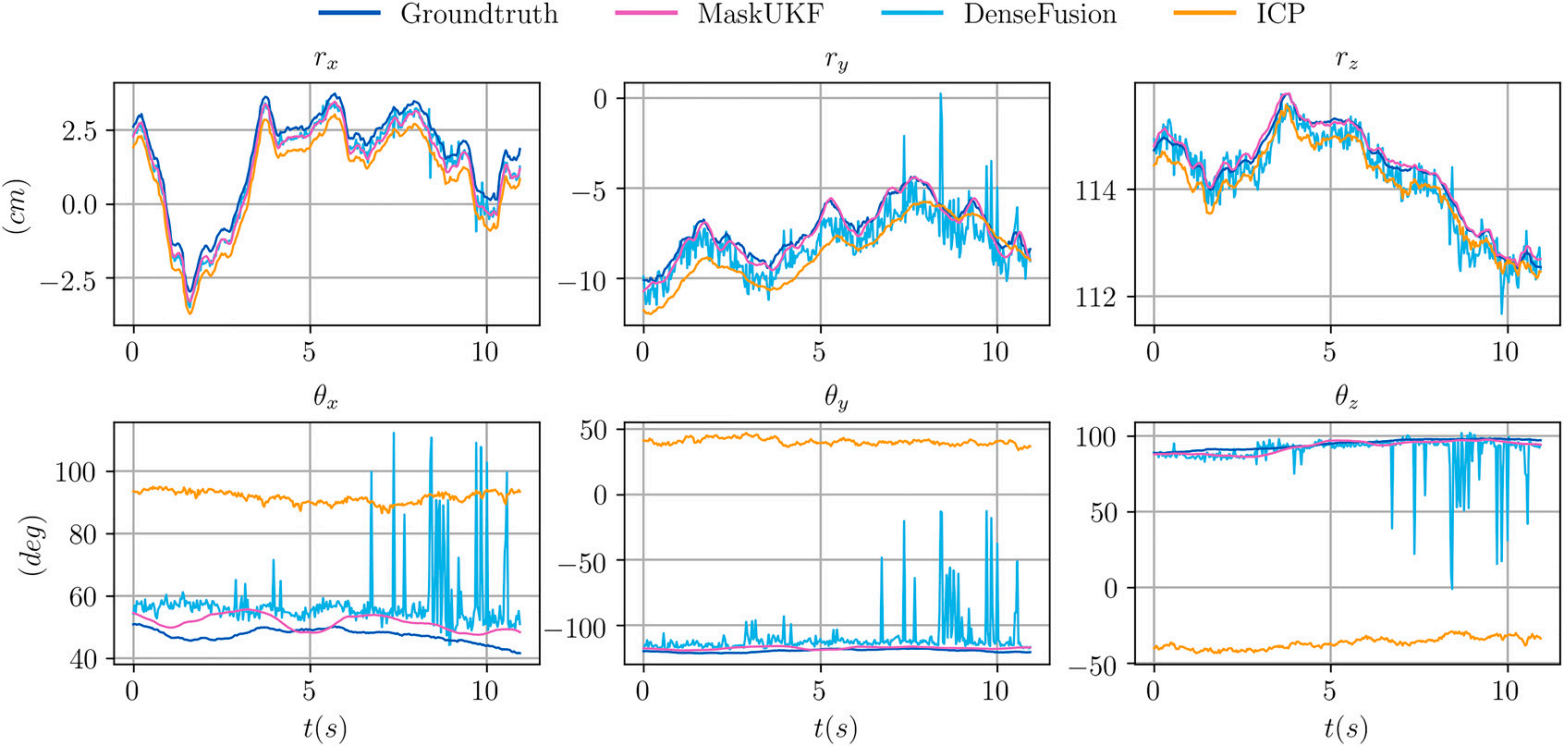
Comparison of the trajectories of several algorithms for the object 021_bleach_cleanser within a subset of the sequence 0055 from the YCB Video Dataset. Our method is more precise than ICP and smoother than DenseFusion which exibits spikes and irregularities. In this figure, the orientation of the object is represented in a compact way using the Euler vector θ=θe obtained as the product of the axis of rotation e and the angle of rotation *θ*.

**FIGURE 3:**
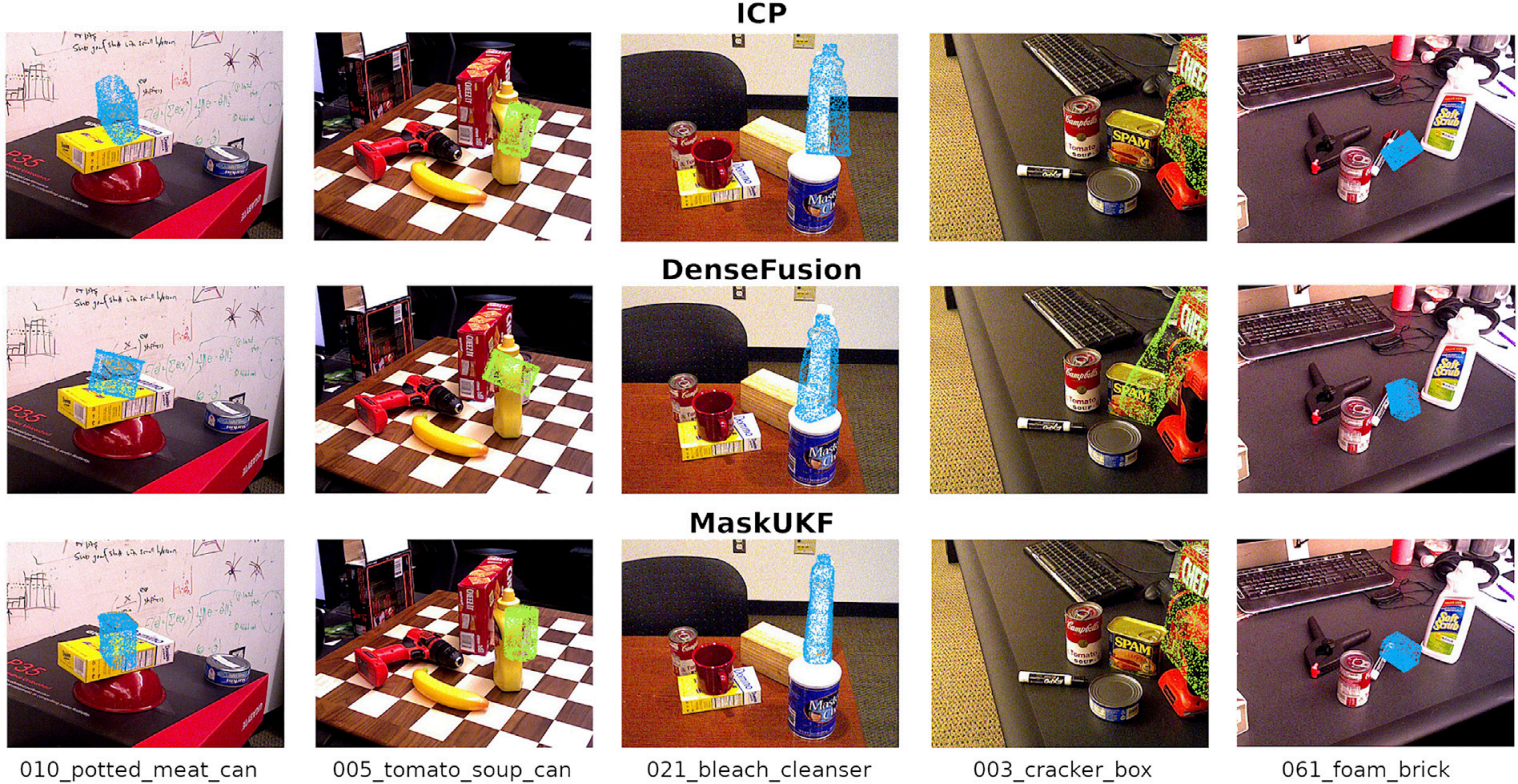
Qualitative results on the YCB Video Dataset. All the results reported here are obtained using the segmentation masks from PoseCNN. The estimated pose is represented as a coloured point cloud transformed in the estimated pose and projected to the 2D RGB frame.

In order to provide an insight on the motivations behind the performance improvement, we provide in Figure 2 an excerpt of the trajectories of the estimated pose for the object 021 in the sequence 0055 according to several algorithms. In that sequence, the object is visible from its shortest and texture-less side (Figure 3) hence producing partial and ambiguous measurements. As can be seen both from the plot and in Figure 3, the estimate from ICP, that uses only depth information, pivots around the measurements resulting in a totally wrong estimate of the orientation. The output orientation from DenseFusion is often wrong despite the availability of RGB information because the visible part of the object is almost texture-less, hence ambiguous. Our algorithm, instead, provides a sound estimate of the pose over time. This can be explained in terms of the motion model in Eq. 28 that helps regularize the estimate when only partial 3D measurements are available using the information encoded in the estimate of the previous step and in the estimated velocities. In the specific case of the sequence 0055, the camera is rotating very slowly around the object of interest, hence the estimated velocities helps enforcing the correct estimate and reducing pivoting phenomena as seen in the outcome of the ICP algorithm. A similar reasoning justifies the performance of our method in presence of occlusions.

#### 4.3.5 Qualitative Evaluation

Figure 3 illustrates some samples 6D estimates on the YCB Video Dataset. As can be seen, both ICP and DenseFusion fail to estimate the correct pose of the cans in the leftmost columns due to challenging occlusion. In the central column, the bleach cleanser is visible from its shortest and texture-less side resulting in wrong estimates of the orientation when using DenseFusion and ICP. Another challenging case is that of the cracker box, on the right, that is only partially visible in the RGB frame. While DenseFusion struggles to estimate the correct orientation, our algorithm and ICP are able to recover it properly. We do not report qualitative results for PoseRBPF as their implementation is not publicly available, hence it is not possible to reproduce the estimated poses.

Figure 2 shows trajectory samples of the estimated pose for one of the object from the YCB Video Dataset obtained by different algorithms. Our algorithm is visibly more precise than ICP and smoother than DenseFusion, which makes it more suitable for closing the control loop in robotic applications.

#### 4.3.6 Multi-Rate Experiment

In Table 4, we report the results of the experiment in which we feed the UKF and the ICP algorithm with masks from Mask R-CNN (He et al., 2017) at the maximum frame rate declared by the authors, i.e., 5 fps. As described in Section 3.6, if the mask at time *t* is not available, our algorithm uses the most recent one instead. In order to make the comparison fair, we adopted the same strategy for the ICP algorithm. We provide the ADD-S (AUC) and RMSE metrics.

**TABLE 4:** Quantitative evaluation of 6D pose on multi-rate scenario using YCB-Video Dataset (ADD-S (AUC), RMSE). Objects in bold are considered symmetric. A bold entry indicates a strictly better result (i.e. if different algorithms have the same best result, the associated entries are not bolded).

	ICP			MaskUKF		
Segm.	Mask R-CNN @ 5 fps					
Objects	AUC	$e_{r}\;\left(\text{cm}\right)$	$e_{o}\;\left({}^{\circ}\right)$	AUC	$e_{r}\;\left(\text{cm}\right)$	$e_{o}\;\left({}^{\circ}\right)$
**002**	93.4	1.29	28.1	**96.0**	**0.603**	**1.61**
003	**88.0**	**3.21**	100	79.0	5.27	**64.2**
004	97.0	**0.624**	76.5	**97.7**	0.779	**2.98**
**005**	93.4	**1.87**	36.1	**95.4**	12.3	**21.7**
006	97.6	0.398	47	**98.1**	**0.353**	**3.48**
**007**	**96.9**	**0.584**	61.1	95.9	0.908	**35.3**
008	**79.6**	**3.95**	158	70.6	13.5	**109**
009	98.4	**0.419**	3.09	**98.5**	0.451	**1.98**
010	**87.9**	3.97	85	84.7	**3.89**	**10.1**
011	95.7	1.47	44.9	**97.5**	**0.974**	**5.39**
019	**97.4**	**0.282**	**2.63**	96.3	0.893	4.04
021	86.9	3.88	44.9	**96.8**	**0.558**	**7.56**
**024**	94.4	1.34	24.4	**97.5**	**0.485**	**3.09**
025	94.6	**0.496**	111	**97.0**	0.536	**35.1**
035	**97.1**	**0.733**	**3.51**	92.7	2.2	15.7
**036**	92.2	2.08	5.19	**94.3**	**1.86**	**2.01**
037	86.2	3.3	29.6	**95.8**	**0.977**	**5.71**
**040**	90.6	2.89	45.4	**95.9**	**1.69**	**6.68**
**051**	42.8	17.8	75.6	**46.7**	**16**	**64.8**
**052**	31.0	16.2	75.4	**37.6**	**13.6**	**53.3**
061	**97.0**	1.8	178	96.9	**0.78**	**11.7**
ALL	87.6	**5.36**	66.6	**88.6**	6.39	**31.2**

In this scenario, our algorithm outperforms the ICP baseline especially in terms of the orientation of the objects. A direct comparison with the results in Table 1 reveals a drop in the ADD-S performance (slightly more accentuated for ICP). This is an expected behavior due to the re-use of old masks on several consecutive frames. The same considerations apply for the position and orientation of most objects except for those, such as 051 and 052, whose masks provided by Mask R-CNN are wrong or missing in several frames. Indeed, if the masks are provided less frequently, there are less chances that the tracking algorithm uses erroneous 3D information extracted from wrong masks.

#### 4.3.7 Results on Velocity Estimation

**FIGURE 4:**
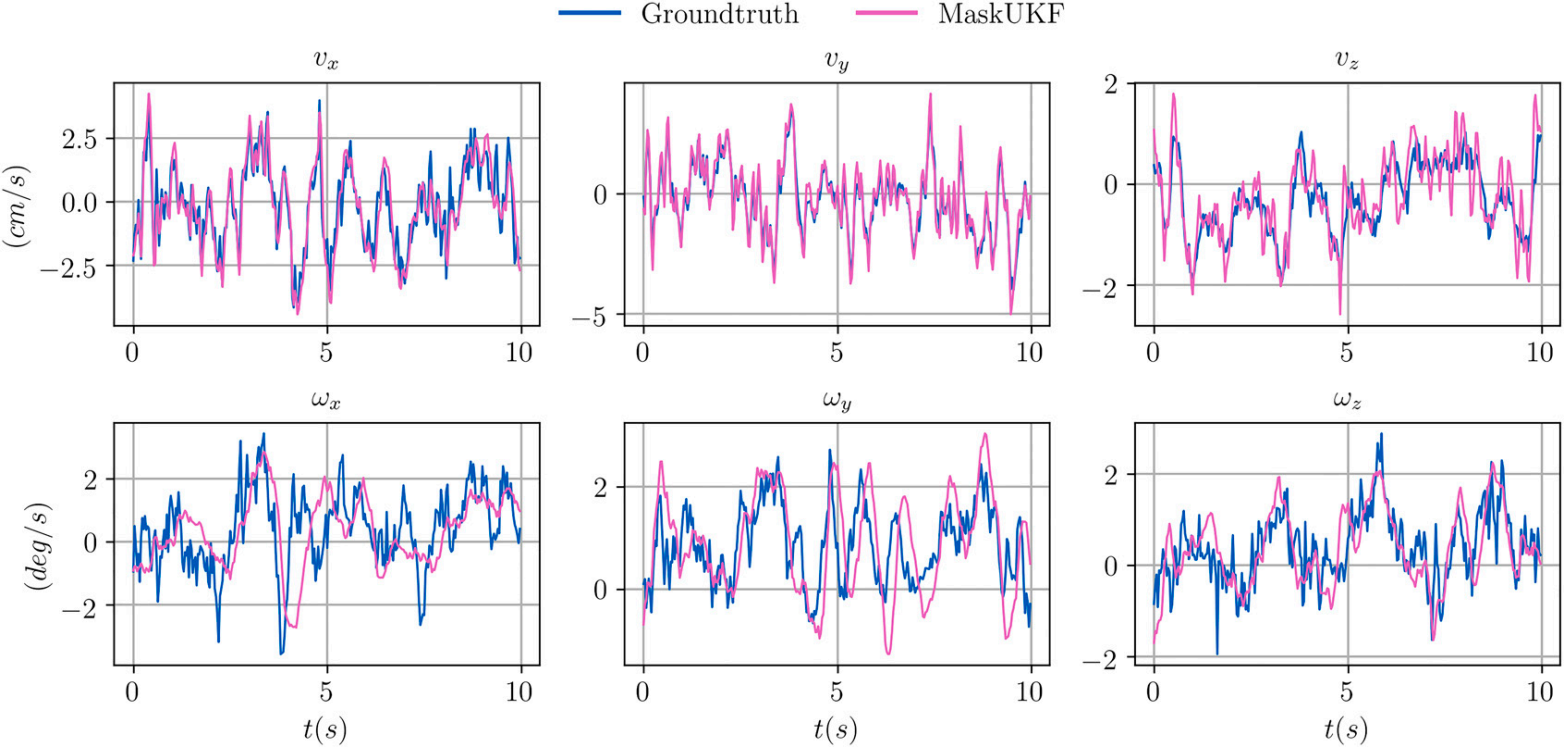
Comparison between estimated linear (*v*) and angular (*ω*) velocities for several algorithms within a video sequence from the YCB Video dataset. The ground truth velocities are obtained from finite differences.

We report a qualitative evaluation of the velocity estimates (linear and angular) in Figure 4, where it is shown that tracking is performed reliably. Given that the YCB Video Dataset does not provide a ground truth for the velocities, we extracted it from the ground truth poses of consecutive frames by means of finite differences. The angular velocity, indicated as *ω*, was evaluated starting from our estimate of the Euler angular rates $\dot{o}$ using the Euler kinematical equation (Stevens et al., 2015).

#### 4.3.8 Effect of Outlier Rejection

Table 5 shows the effectiveness of the outlier rejection procedure presented in Section 3.4. Our algorithm performs better when the outliers are taken into account, especially in terms of reduced angular error.

**TABLE 5:** Effect of the outlier rejection procedure on the performance of MaskUKF with PoseCNN segmentation (averaged ADD-S and RMSE). A bold entry indicates a strictly better result (i.e. if different algorithms have the same best result, the associated entries are not bolded).

Segmentation	PoseCNN			
	AUC	<2 cm	$e_{r}\;\left(\text{cm}\right)$	$e_{o}\;\left({}^{\circ}\right)$
W/outlier rejection	**94.2**	**95.9**	**3.07**	**26**
W/o outlier rejection	83.3	68.9	5.05	76.9

#### 4.3.9 Frame Rate Comparison

We compare our frame rate against DenseFusion and PoseRBPF in Table 6. For our algorithm, we evaluated the frame rate as the inverse of the mean time, averaged on the total number of frames of the testing sequences, required to perform outlier rejection and the Kalman prediction and correction steps. We did not consider the time required to segment the object in our evaluation since, as described in Section 3.6, our method can run asynchronously with respect to the frame rate of the segmentation algorithm. For ICP, the evaluation was done on the mean time required to perform the registration step between the source and target point cloud. For DenseFusion and PoseRBPF we considered the frame rates reported in the associated publications. We notice that the frame rate reported by the authors of DenseFusion, i.e., 16.7 fps, includes the time required to segment the object. In order to have a fair comparison, we omitted the segmentation time resulting in a frame rate of 30 fps for this algorithm.

**TABLE 6:** Frame rate comparison (fps). Our method is approximately 1.5× faster than DenseFusion and 10× faster than PoseRBPF.

DenseFusion (iterative)	PoseRBPF (200 particles)	ICP	MaskUKF
30.0	5.0	91.7	52.6

We ran our experiments on an Intel i7-9750H multi-core CPU. Our method is approximately one and a half times faster than DenseFusion and ten times faster than PoseRBPF. Given its simplicity, the ICP implementation that we adopted reaches 91.7 fps.

### 4.4 Results on Closed Loop Control

**FIGURE 5:**
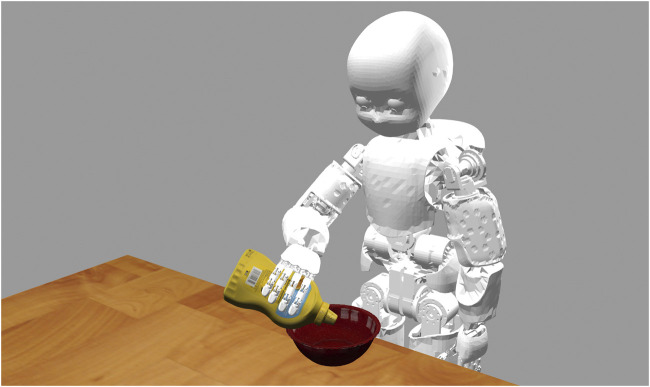
The iCub robot in the Gazebo environment.

In this section we compare our method with DenseFusion and the baseline ICP within a *simulated* robotic experiment where the output of each method is fed as a reference signal to a closed loop control system. For our experiments, we adopt the iCub humanoid robotic platform (Metta et al., 2010) that we simulated in the Gazebo (Koenig and Howard, 2004) environment (Figure 5). We do not test against PoseRBPF since the implementation of the algorithm is not publicly available. Our results include the end-effector tracking errors for several configurations of the control gains in order to compare the algorithms on the tracking precision and reliability when the amount of feedback given to the control system is varied. We also show the qualitative evolution of the end-effector trajectories over time in order to discuss the feasibility of the commanded trajectories.

#### 4.4.1 Description of the Experiment

**FIGURE 6:**
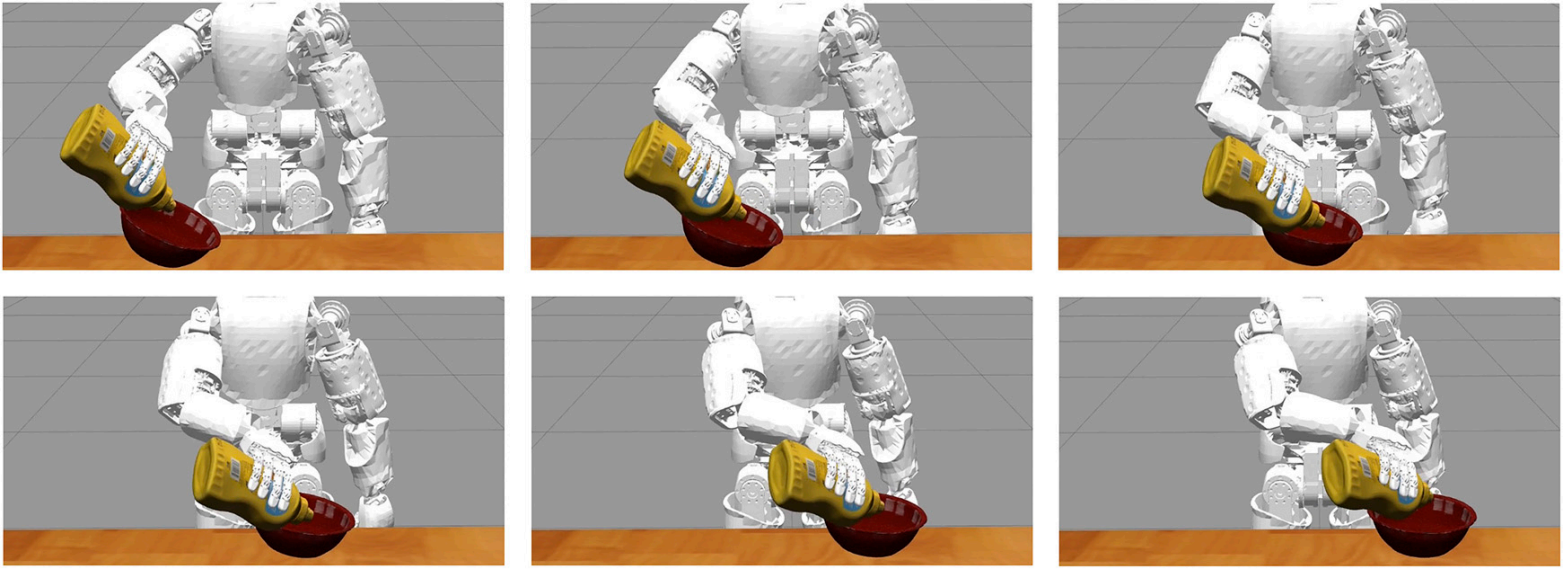
The iCub robot in the Gazebo environment while it follows a moving container (red bowl) during a pouring task using the estimate of the pose and velocity of the container as feedback signal.

In our experiment we consider the task of following a moving container with the end-effector of a humanoid robot while the robot is holding a bottle and pouring its content inside the container. The task is depicted in Figure 6. We assume that the 6D pose and the *velocity* of the container are estimated/tracked using RGB-D information, while we assume that the position of the bottle is known given the robot kinematics (i.e., we focus on the task of tracking the container only). We are not directly interested in the grasping task nor in the pouring action per se but more on the possibility of exploiting the estimated signal in a closed loop fashion in order to follow the container as close as possible while avoiding contacts that might compromise the pouring action. We adopted the YCB Object and Model Set (Calli et al., 2015) for the objects of the experiment by choosing the object 006_mustard_bottle as the bottle and the object 024_bowl as the container (Figure 5).

#### 4.4.2 Implementation of the Experiment

We implemented our experiment in the Gazebo environment using a simulated model of the iCub robotic platform (Metta et al., 2010). Even though iCub features 53 DoF, only a subset of these are used in our experiment, i.e., three DoF in the torso, seven DoF in the arm and six DoF in the head, where the stereo vision system is mounted.

In our experiment we assume that the vision system of the robot provides segmentation and RGB-D information that we use to estimate and/or track the pose of the container. Additionally, we use the iCub gaze control system (Roncone et al., 2016) to track the container in the image plane by using the estimated Cartesian position of the object as a reference gazing point.

In order to carry out the task described in Section 4.4.1, we consider the torso (two DoF out of three) and one of the arms as a nine DoF serial chain whose dynamic behavior is described by the standard equation

$$M\left(q\right)\ddot{q}+h\left(q,\dot{q}\right)=\tau ,$$ (30)

where *q*, $\dot{q}$ and $\ddot{q}\in {\mathbb{R}}^{9}$ are the joints angles, velocities and accelerations, respectively, $M\left(q\right)\in {\mathbb{R}}^{9\times 9}$ is the Mass matrix of the chain, $h\left(q,\dot{q}\right)\in {\mathbb{R}}^{9}$ represents the effect of centrifugal, Coriolis and gravity terms and $\tau \in {\mathbb{R}}^{9}$ are the torques applied to the chain joints axes.

In order to command the end-effector of the robot and follow the moving container over time, we adopted a two-layer control architecture. The first layer consists in an Inverse Dynamics controller

$$\begin{array}{l} \tau_{cmd}=J_{ee}{\left(q\right)}^{T}\text{Λ}\left(q\right)\left({\dot{V}}_{des}-{\dot{J}}_{ee}\left(q\right)\dot{q}\right)+h\left(q,\dot{q}\right),\\ \text{Λ}\left(q\right)={\left(J_{ee}\left(q\right)M{\left(q\right)}^{-1}J_{ee}{\left(q\right)}^{T}\right)}^{-1}. \end{array}$$ (31)

Here, $J_{ee}\in {\mathbb{R}}^{6\times 9}$ is the Jacobian of the end-effector expressed in the robot root frame which links the joints velocities $\dot{q}$ with the end-effector linear velocity $v_{ee}\in {\mathbb{R}}^{3}$ and angular velocity $\omega_{ee}\in {\mathbb{R}}^{3}$

$$\left(\begin{array}{c} v_{ee}\\ \omega_{ee} \end{array}\right)=J_{ee}\left(q\right)\dot{q},$$ (32)

both expressed in the robot root frame. The matrix $\text{Λ}\left(q\right)\in {\mathbb{R}}^{9\times 9}$ in Eq. 31 is also called the Task Space Mass matrix. The term ${\dot{V}}_{des}\in {\mathbb{R}}^{6}$ is a vector containing the desired linear acceleration of the end-effector ${\dot{v}}_{des}\in {\mathbb{R}}^{3}$ and the desired angular acceleration ${\dot{\omega }}_{des}\in {\mathbb{R}}^{3}$

$${\dot{V}}_{des}=\left(\begin{array}{c} {\dot{v}}_{des}\\ {\dot{\omega }}_{des} \end{array}\right),$$ (33)

both expressed in the robot root frame. If controlled using the torques in Eq. 31, the system in Eq. 30 reduces to the system of equations

$${\dot{V}}_{ee}=\left(\begin{array}{c} {\dot{v}}_{ee}\\ {\dot{\omega }}_{ee} \end{array}\right)={\dot{V}}_{des},$$ (34)

where ${\dot{v}}_{ee}\in {\mathbb{R}}^{3}$ is the linear acceleration of the end-effector and ${\dot{\omega }}_{ee}\in {\mathbb{R}}^{3}$ is the angular acceleration. In essence, the first layer allows reducing the dynamics of the serial chain to a linear system having ${\dot{V}}_{des}$ as input.

The second control layer consists in a Proportional Derivative controller

$${\dot{V}}_{des}=k_{p}e_{p}+k_{d}e_{v},$$ (35)

where $e_{p}\in {\mathbb{R}}^{6}$ is the end-effector configuration error

$$e_{p}=\left(\begin{array}{c} p_{ee}-p_{des}\\ \text{log}\left(R_{des}R_{ee}^{T}\right) \end{array}\right),$$ (36)

and $e_{v}\in {\mathbb{R}}^{6}$ is the end-effector velocity error

$$e_{v}=\left(\begin{array}{c} v_{ee}-v_{des}\\ \omega_{ee}-\omega_{des} \end{array}\right).$$ (37)

Here, $p_{ee}\in {\mathbb{R}}^{3}$ is the Cartesian position of the end-effector, $R_{ee}\in SO\left(3\right)$ is the orientation of the end-effector, $v_{ee}\in {\mathbb{R}}^{3}$ is the linear velocity of the end-effector and $\omega_{ee}\in {\mathbb{R}}^{3}$ is the angular velocity that are known via the robot forward kinematics map and forward differential kinematics map. Moreover, $p_{des}\in {\mathbb{R}}^{3}$ is the desired position of the end-effector, $R_{des}\in SO\left(3\right)$ is the desired orientation of the end-effector, $v_{des}\in {\mathbb{R}}^{3}$ is the desired linear velocity of the end-effector and $\omega_{des}\in {\mathbb{R}}^{3}$ is the desired angular velocity. We recall that the expression $\text{log}\left(R_{des}R_{ee}^{T}\right)$ in Eq. 36 represents a proper representation of the angular error in the Lie algebra so(3) (Bullo and Murray, 1999).

In our experiment, we assume that position of the tip of the bottle with respect to the robot end-effector is known at any time. Given this assumption, we consider the end-effector frame ee in Eqs. 36 and 37 to be a frame attached to the tip of the bottle. Given this choice, we conclude the design of our control system by setting the desired quantities as follows:

$$\begin{array}{l} p_{des}=r_{t},\\ R_{des}=R_{des}\left(R_{0},o_{t}\right),\\ v_{des}=v_{t},\\ \omega_{des}=\omega_{t}\left(o_{t},{\dot{o}}_{t}\right), \end{array}$$ (38)

where $r_{t}$, $o_{t}$ are the *estimated* container position and orientation and $v_{t}$, ${\dot{o}}_{t}$ are the *estimated* container Cartesian velocity and the Euler angular rates as defined in Eq. 1. The term $R_{des}\left(R_{0},o_{t}\right)$ takes into account the orientation of the container and a default pouring configuration $R_{0}$ in order to provide the desired orientation of the end-effector while the term $\omega_{t}\left(o_{t},{\dot{o}}_{t}\right)$ represents the conversion from Euler rates to angular velocities.

We remark that the Cartesian velocity and the angular velocity of the container are directly provided by our method as part of the state defined in Eq. 1. In order to execute the experiment with the pose estimation algorithm DenseFusion and with the baseline ICP, we approximated the Cartesian and angular velocity using finite differences.

#### 4.4.3 Evaluation Metrics

In order to compare different algorithms we use the Cartesian error

$$e_{x}=\left|\left|p_{ee,x}-r_{t,x}^{gt}\right|\right|,$$ (39)

between a given coordinate *x* of the end-effector 3D position and the real coordinate *x* of the 3D position of the object $r_{t,x}^{gt}$ and the geodesic angular error (Huynh, 2009)

$$e_{R}=\left|\left|\text{log}\left(R_{des}\left(R_{0},o_{t}^{gt}\right)R_{ee}^{T}\right)\right|\right|,$$ (40)

between the orientation $R_{ee}$ of the end-effector and the desired orientation $R_{des}\left(R_{0},o_{t}^{gt}\right)$ evaluated on the real orientation of the object $o_{t}^{gt}$.

#### 4.4.4 Results of the Experiment

We tested our method, DenseFusion and the baseline ICP on the closed loop experiment described above under the following assumptions:

• a sinusoidal trajectory is assigned to the moving container along the *y* direction of the robot root frame;
• a sinusoidal trajectory is assigned to the orientation of the container along one of its axis;
• before starting the experiment, the end-effector is reset to a rest configuration near the container;
• each experiment lasts 1 min in order to test the reliability of system;
• our method and ICP are initialized using the ground truth from the simulator.

An excerpt of the trajectory of the moving container can be seen in Figure 6.

**FIGURE 7:**
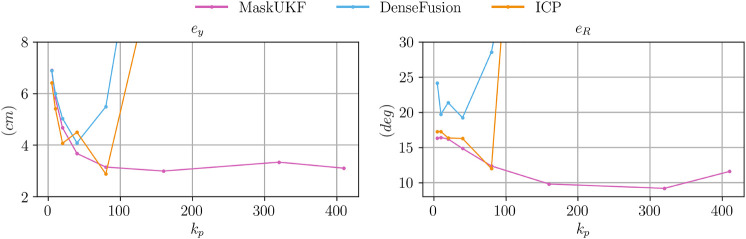
Comparison between RMSE Cartesian and angular error for the object following experiment for varying proportional gains $k_{p}$. Our method allows increasing the gain up to $k_{p}=320$ reaching better tracking performance.

In Figure 7 we compare the three algorithms in terms of Cartesian error $e_{y}$ and the angular error $e_{R}$. Specifically, we consider the Root Mean Square Error (RMSE) along 1 min of experiment for several choices of the proportional gain. For each choice of the gain we assigned the derivative gain $k_{d}$ as $2\sqrt{k_{p}}$ as this choice assures the fastest possible closed loop dynamics for the double integrator system in Eq. 34.

As can be seen from Figure 7, both the errors decrease for all the algorithms when the gain increases up to kp≈50. For higher gains, the error increases for both DenseFusion and ICP resulting in a failure in the experiment for kp=160. Conversely, our method allows increasing the gain up to kp=320 and reaching a lower tracking error especially for the orientation part. This behavior can be explained in terms of the smoothness of the pose estimates produced by our method as we discussed in Section 4.3.5. Furthermore, our method naturally provides a smooth estimate of the velocity of the object, as shown in Section 4.3.7, while for the other algorithms we resorted to velocities obtained by finite differences. Finite difference approximations are typically noisy, even in the case of DenseFusion (whose pose estimates are quite precise but noisy, see Figure 2), thus reducing the maximum possible amount of feedback hence the tracking performance of the closed loop system.

We conclude our analysis by showing the actual trajectories of the end-effector for two specific choices of $k_{p}$, namely 80 and 160.

**FIGURE 8:**
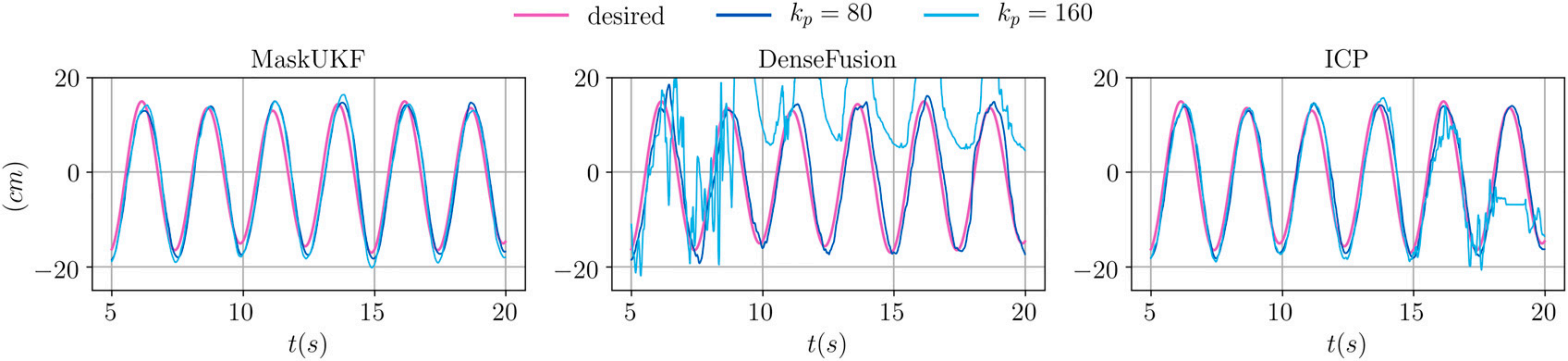
Evolution of the *y* coordinate of the end-effector for several algorithms and different proportional gains.

In Figure 8, we show the desired and achieved trajectory of the *y* coordinate for the first 20 s. As can be seen in the case of $k_{p}=160$, with our method the end-effector achieves a steady and smooth behavior. When using ICP, the end-effector fails to track the container after ≈17 s and shows a moderately noisy behavior. With DenseFusion, tracking is lost after ≈10 s and the motion is characterized by non-neglibile spikes which make it unsafe for the robot.

**FIGURE 9:**
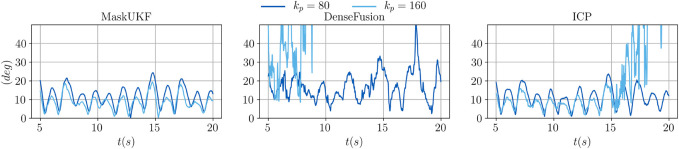
Evolution of the angular error of the end-effector for several algorithms and different proportional gains.

In Figure 9, we show the evolution of the angular error over time for the same choice of the proportional gain. As can be seen, moving from $k_{p}=80$ to $k_{p}=160$ helps reducing the mean angular error when using our method and ICP. However, as already seen for the *y* coordinate, with ICP tracking is lost at some point and the error diverges. When using DenseFusion with $k_{p}=80$, the error is much higher than with MaskUKF and ICP and it diverges when moving to $k_{p}=160$.

In summary, our method provides better performance in terms of tracking precision and reliability when the amount of feedback given to the control system is increased. We stress that this result depends in large part on the adoption of a Kalman filtering framework for our method which, leveraging even a rather simple motion model as in Eq. 28, can produce smooth estimates of both object pose and velocity.

## 5 Conclusion

We proposed MaskUKF, an approach that combines deep instance segmentation with an efficient Unscented Kalman Filter to track the 6D pose and the velocity of an object from RGB-D images. We showed that MaskUKF achieves, and in most cases surpasses, state-of-the-art performance on the YCB video pose estimation benchmark, while providing smooth velocity estimation without the need for expensive pose annotation at training time.

Experiments with the state-of-the-art segmentation algorithm Mask R-CNN at relatively low frame rate of 5 fps suggest possible future research on the integration with recent architectures such as SiamMask (Wang Q. et al., 2019) or Siamese Mask R-CNN (Michaelis et al., 2018) that have proven to provide comparable accuracy at higher frame rates.

Our results show that for some objects, simple solutions like ICP, that operates without a motion model, perform very similarly to the state of the art. This seems to suggest that the YCB Video dataset is not challenging enough, despite having become a popular dataset for pose estimation benchmarking. As future work, we propose to develop more challenging pose estimation and tracking datasets that can effectively show the shortcomings of classical approaches as ICP and motivate the necessity for complex deep learning-based architectures.

Experiments in a simulated dynamical environment highlights superior performance of our method for closed loop control tasks on robotic platforms. At the same time, they show how state-of-the-art RGB-D deep approches, while being precise enough for pose estimation purposes, might be inadequate for control tasks due to unregulated noise in the output which limits the overall performance. As a future work, we propose to develop standard benchmarks, specifically tailored to the robotic community, to ascertain the actual usability of pose estimation/tracking algorithms for such tasks.

## Data Availability

Publicly available datasets were analyzed in this study. This data can be found here: https://rse-lab.cs.washington.edu/projects/posecnn/.

## Footnotes

1
https://github.com/robotology/mask-ukf
